# Burnout and work-engagement profiles, career resources, and psychological distress among early-career doctoral healthcare professionals: a cross-sectional latent profile and network analysis

**DOI:** 10.3389/fpubh.2026.1953465

**Published:** 2026-09-23

**Authors:** Shaobo Liu, Jing He

**Affiliations:** 1 Human Resources Department, Sichuan Provincial People’s Hospital, School of Medicine, University of Electronic Science and Technology of China, Chengdu, China; 2 Department of Nursing, Sichuan Provincial People’s Hospital, School of Medicine, University of Electronic Science and Technology of China, Chengdu, China

**Keywords:** anxiety, career development, depression, healthcare workforce, latent profile analysis, network analysis, occupational burnout, work engagement

## Abstract

**Background:**

Early-career doctoral healthcare professionals combine clinical responsibilities with academic-performance expectations, yet heterogeneity in their burnout and work-engagement patterns and their associations with career resources and psychological distress is poorly characterized.

**Methods:**

We conducted a cross-sectional survey of 386 doctoral healthcare professionals within 3 years of recruitment in Sichuan, China. Burnout dimensions, work engagement, and four career-resource domains were assessed. Anxiety and depressive symptoms were measured using the two-item Generalized Anxiety Disorder scale (GAD-2) and the two-item Patient Health Questionnaire (PHQ-2), respectively. We conducted latent profile analysis using a Gaussian mixture model with a diagonal covariance structure to identify distinct burnout–engagement profiles. Multinomial regression examined profile correlates, robust linear regression compared symptom scores, and a regularized partial-correlation network described conditional associations.

**Results:**

Positive anxiety and depression screens occurred in 79 (20.47%) and 51 (13.21%) participants. A three-profile solution was retained: resilient-engaged (*n* = 65), moderate (*n* = 156), and high-strain (*n* = 165; entropy = 0.92; mean posterior probability = 0.97). Although information criteria decreased through six classes, higher-class solutions accumulated boundary variances or produced classes below 5% under constrained variance. Compared with the resilient-engaged profile, each standard-deviation increase in career-development planning was associated with lower odds of moderate (odds ratio [OR] = 0.26, 95% confidence interval [CI] 0.13–0.52) and high-strain membership (OR = 0.16, 95% CI 0.07–0.36). After adjustment, moderate and high-strain profiles had higher GAD-2 scores by 0.81 and 1.65 points and higher PHQ-2 scores by 0.52 and 1.35 points, respectively. Four-class, posterior-probability, and low-loading-item sensitivity analyses preserved the career-planning association and symptom gradient. Emotional exhaustion was the highest-strength node in 95.90% of bootstrap samples, but strength and bridge-expected-influence stability were moderate (CS = 0.40), and the exact bridge ranking remained scoring-sensitive.

**Conclusion:**

Burnout–engagement profiles followed a severity gradient, with the high-strain profile comprising 42.75% of the sample and showing the greatest psychological distress. Career planning was the most consistent inverse profile correlate, but longitudinal studies are needed before causal or intervention claims can be made.

## Introduction

A stable and psychologically healthy health workforce is essential to resilient health systems and to implementation of the global health-workforce strategy (1, 2). Workforce education and employment policies must also align training with health-system needs (3). Burnout and psychological distress among healthcare professionals are therefore occupational-health and workforce-retention concerns rather than solely individual problems (4, 5). Reviews further connect workforce well-being with career engagement and quality of care (6). Excessive workload is also associated with intention to leave (7). Early-career doctoral healthcare professionals in China occupy a particularly demanding position: hospitals recruit them to deliver clinical services while simultaneously expecting research productivity, grant acquisition, teaching, and rapid professional advancement (5, 8). These overlapping demands can create persistent resource loss during the transition into an independent career.

Conservation of resources (COR) theory proposes that people seek to obtain and protect valued resources, including time, energy, role clarity, and contextual support. Strain is expected when resources are threatened or lost, or when available resources are insufficient to meet demands; resource gains can protect remaining resources and support continued investment (9, 10). In the present study, burnout dimensions were treated as indicators of perceived depletion, work engagement as sustained occupational energy and investment, and career resources as contextual resource reservoirs (11). This mapping provides an interpretive framework rather than a longitudinal test of a COR loss or gain spiral. For early-career doctoral healthcare professionals, credible career pathways, mentoring, research infrastructure, and transparent talent policies may reduce uncertainty about how limited time and effort should be allocated (12, 13). This organizational emphasis is consistent with calls to address healthcare burnout through changes in working conditions rather than individual resilience alone (14).

Person-centered methods can identify such combinations without imposing arbitrary cut-points. Latent profile studies among hospital doctors and emergency nurses have reported heterogeneous burnout configurations (15, 16). A study of physicians assessed around night shifts similarly identified distinct well-being profiles (17). In parallel, network analysis can estimate conditional associations among occupational resources, burnout dimensions, engagement, and symptoms (18, 19). Bridge indices can describe variables connecting prespecified domains (20). Network studies in Chinese physicians further illustrate close connections among burnout, anxiety, and depression (21). Similar patterns have been reported among residents in standardized training (22).

Accordingly, this study aimed to identify burnout–engagement profiles among early-career doctoral healthcare professionals, examine how career resources and psychological symptoms differed across profiles, and explore the network linking career resources, burnout, work engagement, and psychological symptoms. We treated occupational well-being as the joint pattern of burnout, work engagement, anxiety, and depressive symptoms, rather than as a separate measure of happiness or life satisfaction. We hypothesized that higher-strain profiles would have fewer career resources and greater psychological distress. By combining person-centered and network perspectives, the study sought to clarify both subgroup heterogeneity and conditional relationships relevant to early-career professional development.

## Materials and methods

### Study design, setting, and participants

This cross-sectional study was conducted from January to April 2026 in Sichuan Province, China, and is reported in accordance with the Strengthening the Reporting of Observational Studies in Epidemiology (STROBE) statement for cross-sectional studies (23). Participants were healthcare professionals holding a Doctor of Philosophy (PhD), Doctor of Medicine (MD), or equivalent doctoral degree who had been recruited within the previous 3 years, had at least 3 months of clinical or professional tenure, were currently working, and provided electronic informed consent. Individuals on long-term leave or external secondment/training and questionnaires with substantial incompleteness or implausible response patterns were excluded. The Ethics Committee of Sichuan Provincial People’s Hospital approved the study (No. 2026-294). All participants provided electronic informed consent. Procedures complied with the Declaration of Helsinki.

Recruitment combined purposive sampling at a tertiary research-oriented teaching hospital with participant referral to eligible peers at comparable regional hospitals. Questionnaires were distributed through institutional email and work-related WeChat groups. The original export contained 409 responses. Twenty-three respondents working full-time in research-only posts were excluded from the prespecified clinical cohort; accordingly, the questionnaire branch containing the five clinical-capacity items had not been presented to them. The final analysis cohort therefore comprised 386 participants. To protect participant privacy, identifying information, including personal identifiers and Internet Protocol addresses, was masked before analysis.

### Measures

#### Occupational burnout

Occupational burnout was assessed with a study-specific 12-item instrument adapted from the three-dimensional Chinese occupational-burnout framework (24, 25). It comprised emotional exhaustion (4 items), depersonalization (5 items), and professional efficacy (3 items). Each item was rated on a five-point frequency scale from 1 (never) to 5 (every day). The three efficacy items were reverse-scored once so that higher values represented reduced professional efficacy. A summed score would range from 12 to 60, with higher scores indicating greater overall burnout; however, the analyses used dimension-level item means (range 1–5) because the three dimensions served as separate latent-profile and network indicators.

#### Work engagement

Work engagement was assessed with nine adapted items informed by the Utrecht Work Engagement Scale framework (26). Responses ranged from 1 (almost never) to 5 (almost always), and one negatively worded item was reverse-scored. The possible summed score ranged from 9 to 45; the primary analyses used the item mean (range 1–5), with higher scores indicating greater work engagement.

#### Anxiety and depressive symptoms

Anxiety and depressive symptoms were measured using the two-item Generalized Anxiety Disorder scale (GAD-2) and the two-item Patient Health Questionnaire (PHQ-2), respectively (27, 28). Standard response options range from 0 (not at all) to 3 (nearly every day). Because the questionnaire export stored these options as 1–4, responses were recoded to 0–3 before summation. Each scale therefore ranged from 0 to 6, and a score of 3 or higher indicated a positive screen. Scores were analyzed continuously, and positive-screen prevalence was reported descriptively; neither instrument establishes a clinical diagnosis.

#### Career-development resources

A locally developed questionnaire based on literature review and expert consultation assessed perceived organizational resources. The initial 20-item instrument covered research-resource support, clinical-competence enhancement, career-development planning, and talent-policy support. During initial questionnaire refinement, the negatively framed item “Excessive clinical work pressure has affected my capacity enhancement” had a standardized loading below 0.40 in the intended clinical-competence domain and was excluded before scoring and confirmatory factor analysis (CFA). The final questionnaire contained 19 items: 5 research-resource, 4 clinical-competence, 5 career-planning, and 5 talent-policy items. Responses ranged from 1 (strongly disagree/very dissatisfied) to 5 (strongly agree/very satisfied). Three retained negatively worded items (rs5, cp5, and tp5) were reverse-scored so that higher values consistently indicated greater perceived resources. The possible total score ranged from 19 to 95; primary analyses used domain item means (range 1–5). For clinical competence, a person-mean was calculated when at least two of the four retained items were observed. The complete questionnaire items and scoring key are provided in Supplementary Table S15.

#### Job satisfaction and covariates

Job satisfaction was assessed with 20 adapted items informed by the Minnesota Satisfaction Questionnaire (29), covering achievement, pay, promotion, interpersonal relationships, and work environment. Items ranged from 1 (very dissatisfied) to 5 (very satisfied), giving a possible summed score of 20–100; higher scores indicated greater satisfaction. Job satisfaction was summarized descriptively and was not included as a predictor in the prespecified profile model. Demographic variables comprised age, sex, department, degree, professional title, employment type, marital status, and the presence of clinical and research mentors.

### Scale assessment

Internal consistency was summarized with Cronbach’s alpha. In this sample, alpha was 0.94 for emotional exhaustion, 0.94 for depersonalization, 0.81 for reduced professional efficacy, 0.93 for work engagement, 0.88 for GAD-2, 0.85 for PHQ-2, and 0.97 for job satisfaction. For the career-resource domains, alpha was 0.87 for research-resource support, 0.88 for clinical-competence enhancement, 0.83 for career-development planning, and 0.86 for talent-policy support; McDonald’s omega values were 0.91, 0.91, 0.90, and 0.90, respectively (Supplementary Tables S1 and S19A). Maximum-likelihood CFA evaluated the three-factor burnout structure and the four-factor career-resource structure. Burnout showed chi-square/degrees-of-freedom = 3.53, comparative fit index (CFI) = 0.97, Tucker–Lewis index (TLI) = 0.96, and root mean square error of approximation (RMSEA) = 0.08; standardized loadings ranged from 0.72 to 0.94. The career-resource model showed chi-square/degrees-of-freedom = 3.92, CFI = 0.92, TLI = 0.91, and RMSEA = 0.09; standardized loadings ranged from 0.35 to 0.90 (Supplementary Tables S2, S3). An ordinal polychoric diagonally weighted least-squares sensitivity model showed improved global fit (CFI = 1.00, TLI = 1.00, RMSEA = 0.00), but low item loadings persisted and two heterotrait–monotrait ratios exceeded 0.90, indicating limited separation of some resource domains (Supplementary Tables S19A and S19B). These estimates describe internal structure in the present sample and do not constitute external validation.

Low-loading-item sensitivity analysis. Within the final 19-item career-resource questionnaire, the retained research-resource item rs5 and career-planning item cp5 had standardized loadings of 0.38 and 0.35, respectively. They were retained because prespecified content coverage was considered alongside loading magnitude, rather than applying an automatic *post hoc* deletion rule. Sensitivity analyses excluded rs5 and cp5 separately and jointly, recalculated the relevant domain scores, and refitted the four-factor CFA, profile-regression model, and regularized network. These analyses were conducted only to assess robustness; all primary results were based on the 19-item questionnaire.

### Statistical analysis

#### Latent profile analysis

Emotional exhaustion, depersonalization, reduced professional efficacy, and work engagement were standardized and entered into Gaussian mixture models. The primary specification allowed diagonal indicator variances to differ by class; one- to six-class candidates were each refitted with 500 random initializations and a variance lower bound of 1e-6. We reported log likelihood, Akaike information criterion (AIC), Bayesian information criterion (BIC), sample-size-adjusted Bayesian information criterion (SABIC), entropy, mean maximum posterior probability, smallest class size, and the number of variance estimates at the lower bound. Entropy and mean maximum posterior probability of at least 0.80 and a smallest class of at least 5% and 20 participants were used as practical decision aids, not as formal hypothesis tests. The retained solution was selected from the joint evidence on fit trajectory, boundary estimates, class size, stability, parsimony, and substantive interpretability (30). Equal-diagonal-variance two- to six-class models were fitted with 200 random starts, and four-class profile and outcome models were examined as specification sensitivities. The three-class solution was retained because it was the first stable and interpretable low/moderate/high-strain solution meeting the classification and size criteria; higher-class solutions either accumulated boundary variances or created classes below 5% under the constrained-variance specification Supplementary Tables S8–S11, S16–S18).

#### Regression analyses

Continuous variables were summarized using the mean and standard deviation (SD), and categorical variables using counts and percentages. Kruskal–Wallis and chi-square tests described unadjusted differences but were not used to select covariates. Associations were reported as odds ratios (ORs) with 95% confidence intervals (CIs). A prespecified multinomial logistic regression used the resilient-engaged profile as the reference and simultaneously included standardized age, sex, standardized scores for the four career-resource domains, clinical and research mentoring, and department. Heteroskedasticity-consistent type 0 (HC0) standard errors were used, and variance-inflation factors assessed collinearity. To propagate classification uncertainty, 500 pseudo-class datasets were generated by sampling each participant’s class from the fitted posterior-probability vector. The multinomial and distress regressions were refitted in every dataset, and coefficients and standard errors were combined using Rubin’s rules.

GAD-2 and PHQ-2 scores were compared across profiles with ordinary least-squares models using heteroskedasticity-consistent type 3 (HC3) standard errors. Model 1 was unadjusted, model 2 adjusted for age and sex, and model 3 additionally adjusted for department and the four career-resource domains. Positive-screen prevalence was reported descriptively because the resilient-engaged profile contained no positive screens, which produced complete separation for binary regression.

#### Network estimation, accuracy, and comparison

A network was estimated for 10 standardized variables: three burnout dimensions, work engagement, four career-resource domains, GAD-2, and PHQ-2. GraphicalLassoCV selected the regularization parameter by five-fold cross-validation (19). Edges represent regularized partial correlations conditional on all other nodes. Node strength and bridge expected influence were calculated; communities were defined *a priori* as burnout/engagement, career resources, and psychological symptoms (20).

Edge accuracy was examined with 1,000 nonparametric bootstrap resamples. The same resamples were used to quantify pairwise strength differences and the probability that each node occupied the highest-strength or most-negative-bridge position. Strength and bridge-expected-influence stability were evaluated with 200 case-dropping resamples at each retained fraction and summarized with correlation-stability coefficients (31). No generic *post hoc* power threshold was applied because network sample requirements depend on the hypothesized structure, estimator, and performance target (32). An exploratory sex-stratified comparison used 1,000 label permutations for global strength and the maximum edge difference. Two-sided *p* values below 0.05 were considered statistically significant; the network analyses emphasized effect patterns and uncertainty rather than isolated *p*-values. All analyses were rerun from the original questionnaire export in Python 3.12.

## Results

### Population characteristics

The analysis included 386 participants, with a mean age of 31.05 years (SD 2.69); 227 (58.81%) were women (Table 1). Overall, 79 participants (20.47%) screened positive for anxiety and 51 (13.21%) for depression. Internal-consistency estimates ranged from 0.81 for reduced professional efficacy to 0.94 for depersonalization; work engagement had an alpha of 0.93 (Table 2). For the career-resource domains, omega ranged from 0.90 to 0.91. The burnout CFA showed CFI = 0.97, TLI = 0.96, and RMSEA = 0.08. The career-resource CFA showed CFI = 0.92, TLI = 0.91, and RMSEA = 0.09. Although ordinal DWLS sensitivity improved global fit, the low-loading items remained and HTMT was 0.93 for research resources versus talent policy and for career planning versus talent policy, indicating incomplete discriminant validity (Supplementary Tables S19A and S19B). Excluding rs5 and cp5 jointly improved the maximum-likelihood career-resource CFA fit (CFI = 0.94, TLI = 0.93, RMSEA = 0.08); the reduced scores remained highly correlated with their primary scores (research resources *r* = 0.97; career planning *r* = 0.96). Career planning remained associated with lower odds of moderate (OR = 0.32, 95% CI 0.14–0.75; *p* = 0.009) and high-strain membership (OR = 0.28, 95% CI 0.11–0.68; *p* = 0.005) (Supplementary Tables S12, S13).

**Table 1:** Participant characteristics by latent profile.

Variable	Overall *N* = 386	Resilient-engaged *n* = 65	Moderate *n* = 156	High-strain *n* = 165	*p*-value
Age, years	31.05 (2.69)	31.88 (3.25)	30.85 (2.62)	30.92 (2.45)	0.048
Research resources	2.99 (0.98)	4.02 (0.90)	3.09 (0.85)	2.50 (0.78)	<0.001
Clinical competence	3.68 (0.84)	4.48 (0.71)	3.81 (0.68)	3.24 (0.74)	<0.001
Career planning	3.54 (0.74)	4.43 (0.58)	3.62 (0.59)	3.10 (0.57)	<0.001
Talent policy	3.34 (0.85)	4.31 (0.68)	3.44 (0.68)	2.85 (0.68)	<0.001
GAD-2	1.73 (1.45)	0.42 (0.73)	1.47 (1.18)	2.48 (1.45)	<0.001
PHQ-2	1.33 (1.32)	0.22 (0.54)	1.02 (0.90)	2.06 (1.46)	<0.001
Gender					
Male	159 (41.19%)	31 (47.69%)	65 (41.67%)	63 (38.18%)	0.414
Female	227 (58.81%)	34 (52.31%)	91 (58.33%)	102 (61.82%)	0.414
Department					
Surgery	128 (33.16%)	16 (24.62%)	49 (31.41%)	63 (38.18%)	0.004
Other	137 (35.49%)	36 (55.38%)	51 (32.69%)	50 (30.30%)	0.004
Internal medicine	121 (31.35%)	13 (20.00%)	56 (35.90%)	52 (31.52%)	0.004
Degree					
Medical doctorate	344 (89.12%)	57 (87.69%)	137 (87.82%)	150 (90.91%)	0.621
Other doctorate	42 (10.88%)	8 (12.31%)	19 (12.18%)	15 (9.09%)	0.621
Professional title					
Residency training	125 (32.38%)	26 (40.00%)	51 (32.69%)	48 (29.09%)	0.281
Intermediate or senior	117 (30.31%)	22 (33.85%)	43 (27.56%)	52 (31.52%)	0.281
Junior	144 (37.31%)	17 (26.15%)	62 (39.74%)	65 (39.39%)	0.281
Employment					
Permanent	311 (80.57%)	52 (80.00%)	126 (80.77%)	133 (80.61%)	0.991
Other	75 (19.43%)	13 (20.00%)	30 (19.23%)	32 (19.39%)	0.991
Clinical mentor	177 (45.85%)	25 (38.46%)	78 (50.00%)	74 (44.85%)	0.276
Research mentor	136 (35.23%)	26 (40.00%)	60 (38.46%)	50 (30.30%)	0.210
Marital status					
Married	171 (44.30%)	37 (56.92%)	71 (45.51%)	63 (38.18%)	0.033
Single/other	215 (55.70%)	28 (43.08%)	85 (54.49%)	102 (61.82%)	0.033

Values are mean (SD) or *n* (%). *p*-values are descriptive global Kruskal–Wallis or chi-square tests; profile membership was estimated from the same data. SD, standard deviation; GAD-2, two-item Generalized Anxiety Disorder scale; PHQ-2, two-item Patient Health Questionnaire.

**Table 2:** Burnout and engagement indicators by latent profile.

Indicator	Cronbach alpha	Resilient-engaged Mean (SD)	Moderate Mean (SD)	High-strain Mean (SD)
Emotional exhaustion	0.94	1.22 (0.34)	2.41 (0.50)	3.50 (0.63)
Depersonalization	0.94	1.00 (0.00)	1.84 (0.33)	3.00 (0.62)
Reduced professional efficacy	0.81	1.85 (1.29)	2.17 (0.67)	2.67 (0.54)
Work engagement	0.93	4.73 (0.31)	4.02 (0.41)	3.20 (0.55)

All scores are item means on a 1–5 scale. Higher reduced-professional-efficacy values indicate lower perceived efficacy.

### Latent profile selection and characteristics

Information criteria decreased from the one- through six-class class-specific models, but this improvement was accompanied by increasing boundary-level variances: one in the three-class model, two in the four-class model, three in the five-class model, and seven in the six-class model. The six-class solution also contained only five participants (1.30%). Under equal-diagonal-variance constraints, the three-class solution retained adequate classification (entropy = 0.86; smallest class n = 76, 19.69%), whereas the four-, five-, and six-class solutions produced smallest classes of 14 (3.63%), 10 (2.59%), and 1 (0.26%), respectively. The four-class model mainly split the intermediate profile into two adjacent groups with similar emotional-exhaustion and work-engagement means, while the career-planning and symptom gradients were preserved. On this joint evidence, the three-class solution was retained as the most parsimonious substantively distinct solution (Table 3 and Supplementary Tables S8–S11, S16).

**Table 3:** Latent profile model selection.

Classes	AIC	BIC	SABIC	Entropy	Smallest class n	Smallest class %	Retained
1	4397.70	4429.30	4403.90	1.00	386	100.00	No
2	3832.80	3900.00	3846.10	0.82	184	47.67	No
3	2781.10	2884.00	2801.50	0.92	65	16.84	Yes
4	2072.40	2210.80	2099.80	0.96	65	16.84	No
5	1769.50	1943.50	1803.90	0.96	21	5.44	No
6	879.50	1089.20	921.00	0.97	5	1.30	No

Class-specific diagonal-covariance Gaussian mixture models were refitted with 500 random starts and a variance lower bound of 1e-6. Information criteria continued to decrease, but higher-class solutions accumulated boundary-level variances and subdivided adjacent severity or floor-level patterns. Entropy and mean maximum posterior probability of at least 0.80 and a smallest class of at least 5% and 20 participants were practical decision aids; selection also considered stability, parsimony, and interpretability. Full diagnostics are reported in Supplementary Tables S8–S11 and S16. AIC, Akaike information criterion; BIC, Bayesian information criterion; SABIC, sample-size-adjusted BIC.

Figure 1 presents the mean scores of the four burnout–engagement indicators across the three profiles. The resilient–engaged profile comprised 65 participants (16.84%) and was characterized by low emotional exhaustion (mean = 1.22), minimal depersonalization (1.00), relatively low reduced efficacy (1.85), and high engagement (4.73). The moderate profile comprised 156 participants (40.41%) and showed intermediate scores for emotional exhaustion (2.41), depersonalization (1.84), reduced efficacy (2.17), and engagement (4.02). The high-strain profile comprised 165 participants (42.75%) and showed the highest emotional exhaustion (3.50), depersonalization (3.00), and reduced efficacy (2.67), together with the lowest engagement (3.20).

**Figure 1:**
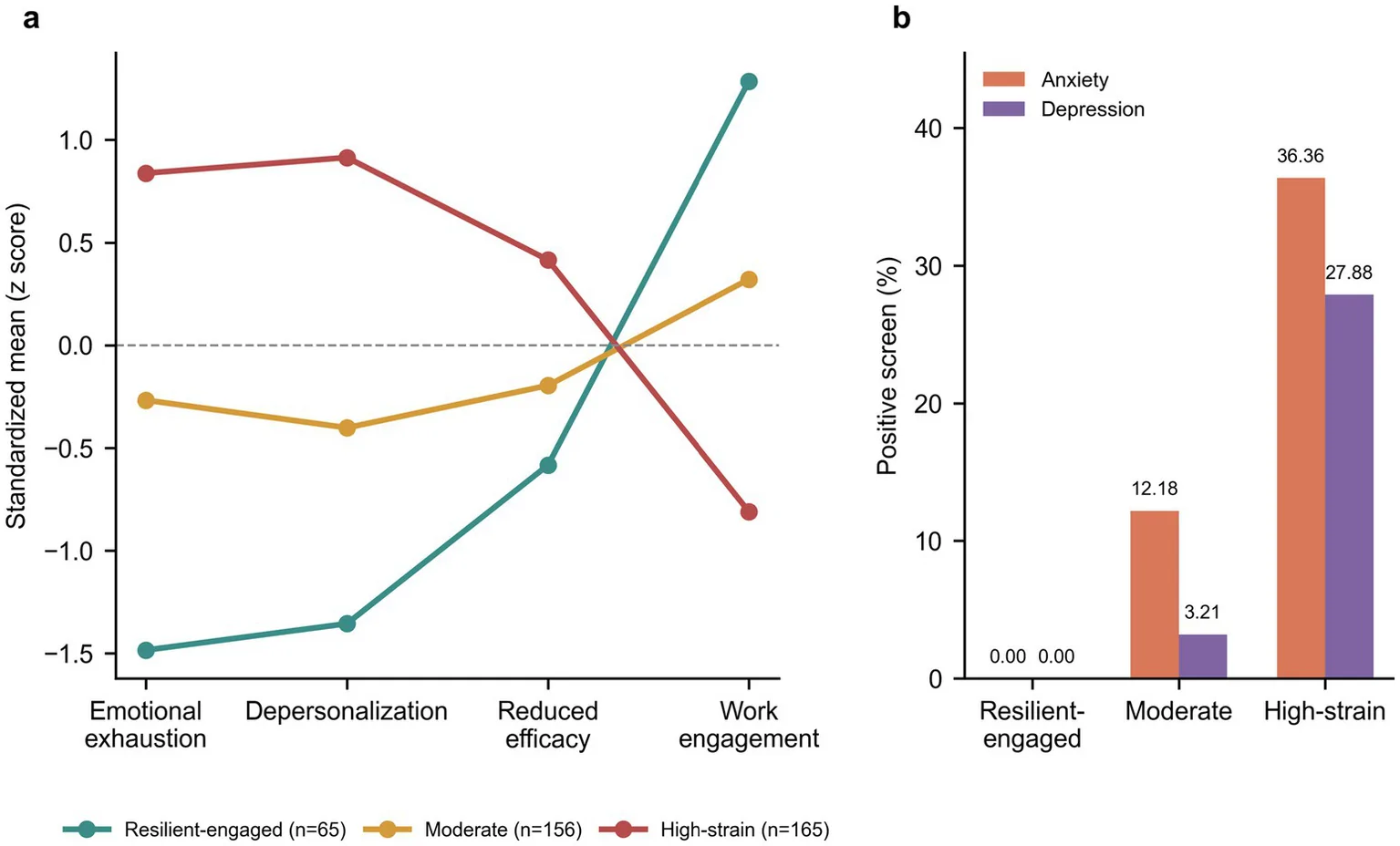
Burnout–engagement profile means and psychological-distress prevalence. Panel **(a)** shows standardized mean scores for the four profile indicators; panel **(b)** shows the percentage screening positive on the two-item Generalized Anxiety Disorder scale (GAD-2) and the two-item Patient Health Questionnaire (PHQ-2) (cutoff ≥3).

### Correlates of profile membership

In the adjusted multinomial model, career-development planning was the most consistent profile correlate. Per standard-deviation increase, the odds of moderate versus resilient-engaged membership were 0.26 (95% CI 0.13–0.52; *p* < 0.001), and the odds of high-strain versus resilient-engaged membership were 0.16 (95% CI 0.07–0.36; *p* < 0.001). Older age was associated with lower odds of both moderate (OR = 0.70, 95% CI 0.50–1.00; *p* = 0.050) and high-strain membership (OR = 0.67, 95% CI 0.46–0.97; *p* = 0.036). Talent-policy support was imprecisely associated with high-strain membership (OR = 0.39, 95% CI 0.15–1.01; *p* = 0.053). Sex, research resources, and mentoring were not independently associated with profile membership. The maximum variance-inflation factor was 4.17. Posterior-probability pseudo-class analysis produced nearly identical career-planning estimates for moderate (OR = 0.26, 95% CI 0.13–0.53; *p* < 0.001) and high-strain membership (OR = 0.16, 95% CI 0.07–0.37; *p* < 0.001); research resources remained non-significant (Table 4 and Supplementary Table S18).

**Table 4:** Adjusted multinomial regression of latent profile membership.

Comparison	Predictor	OR	95% CI	*p*-value
Moderate vs. Resilient-engaged	Age (per SD)	0.70	0.50–1.00	0.050
Moderate vs. Resilient-engaged	Female sex	1.21	0.55–2.66	0.644
Moderate vs. Resilient-engaged	Research resources (per SD)	0.97	0.49–1.92	0.930
Moderate vs. Resilient-engaged	Clinical competence (per SD)	0.82	0.42–1.58	0.545
Moderate vs. Resilient-engaged	Career planning (per SD)	0.26	0.13–0.52	<0.001
Moderate vs. Resilient-engaged	Talent policy (per SD)	0.55	0.25–1.23	0.148
Moderate vs. Resilient-engaged	Clinical mentor: yes	1.84	0.81–4.17	0.147
Moderate vs. Resilient-engaged	Research mentor: yes	1.06	0.47–2.42	0.882
Moderate vs. Resilient-engaged	Department: other vs. internal medicine	0.31	0.13–0.76	0.010
Moderate vs. Resilient-engaged	Department: surgery vs. internal medicine	0.65	0.24–1.79	0.405
High-strain vs. Resilient-engaged	Age (per SD)	0.67	0.46–0.97	0.036
High-strain vs. Resilient-engaged	Female sex	1.42	0.55–3.63	0.466
High-strain vs. Resilient-engaged	Research resources (per SD)	0.75	0.35–1.64	0.478
High-strain vs. Resilient-engaged	Clinical competence (per SD)	0.52	0.25–1.11	0.091
High-strain vs. Resilient-engaged	Career planning (per SD)	0.16	0.07–0.36	<0.001
High-strain vs. Resilient-engaged	Talent policy (per SD)	0.39	0.15–1.01	0.053
High-strain vs. Resilient-engaged	Clinical mentor: yes	1.92	0.76–4.85	0.165
High-strain vs. Resilient-engaged	Research mentor: yes	1.16	0.45–2.98	0.755
High-strain vs. Resilient-engaged	Department: other vs. internal medicine	0.33	0.11–0.98	0.046
High-strain vs. Resilient-engaged	Department: surgery vs. internal medicine	1.09	0.34–3.46	0.886

Reference profile: resilient-engaged. Continuous predictors were standardized. All listed predictors were entered simultaneously. Robust HC0 standard errors were used. Abbreviations: OR, odds ratio; CI, confidence interval; SD, standard deviation; HC0, heteroskedasticity-consistent type 0.

### Profiles and psychological distress

In fully adjusted models, compared with the resilient-engaged profile, GAD-2 scores were 0.81 points higher (95% CI 0.43–1.18; *p* < 0.001) in the moderate profile and 1.65 points higher (95% CI 1.16–2.14; *p* < 0.001) in the high-strain profile. PHQ-2 scores were 0.52 points higher (95% CI 0.20–0.83; *p* = 0.001) and 1.35 points higher (95% CI 0.90–1.80; *p* < 0.001), respectively (Table 5). Positive anxiety screens occurred in 0.00, 12.18, and 36.36% of the resilient-engaged, moderate, and high-strain profiles; positive depression screens occurred in 0.00, 3.21, and 27.88% (Figure 1). After posterior-probability propagation, the corresponding adjusted differences were 0.79 and 1.66 points for GAD-2 and 0.50 and 1.32 points for PHQ-2, with all confidence intervals excluding zero (Supplementary Table S18).

**Table 5:** Fully adjusted robust linear regression of psychological symptoms.

Outcome	Comparison	Beta	95% CI	*P*-value	*R* ^2^
GAD-2	Moderate vs. Resilient-engaged	0.81	0.43–1.18	<0.001	0.29
GAD-2	High-strain vs. Resilient-engaged	1.65	1.16–2.14	<0.001	0.29
PHQ-2	Moderate vs. Resilient-engaged	0.52	0.20–0.83	0.001	0.32
PHQ-2	High-strain vs. Resilient-engaged	1.35	0.90–1.80	<0.001	0.32

Reference profile: resilient-engaged. Model 3 adjusted for age, sex, department, and the four career-resource domains; HC3 robust standard errors were used. Beta is the adjusted mean score difference. Abbreviations: CI, confidence interval; R^2^, coefficient of determination; HC3, heteroskedasticity-consistent type 3; GAD-2, two-item Generalized Anxiety Disorder scale; PHQ-2, two-item Patient Health Questionnaire.

### Network structure and stability

The regularized network selected an alpha of 0.0122. The strongest partial correlations linked anxiety with depression (0.66), emotional exhaustion with depersonalization (0.57), research resources with talent-policy support (0.50), career planning with talent-policy support (0.35), and clinical competence with career planning (0.31). Engagement was negatively connected with emotional exhaustion (−0.29) and reduced efficacy (−0.28) (Figure 2). Pearson correlations among the 10 core variables are shown in Supplementary Figure S1. Detailed node-centrality estimates, edge weights with bootstrap intervals, and case-dropping stability results are provided in Supplementary Tables S4–S6.

**Figure 2:**
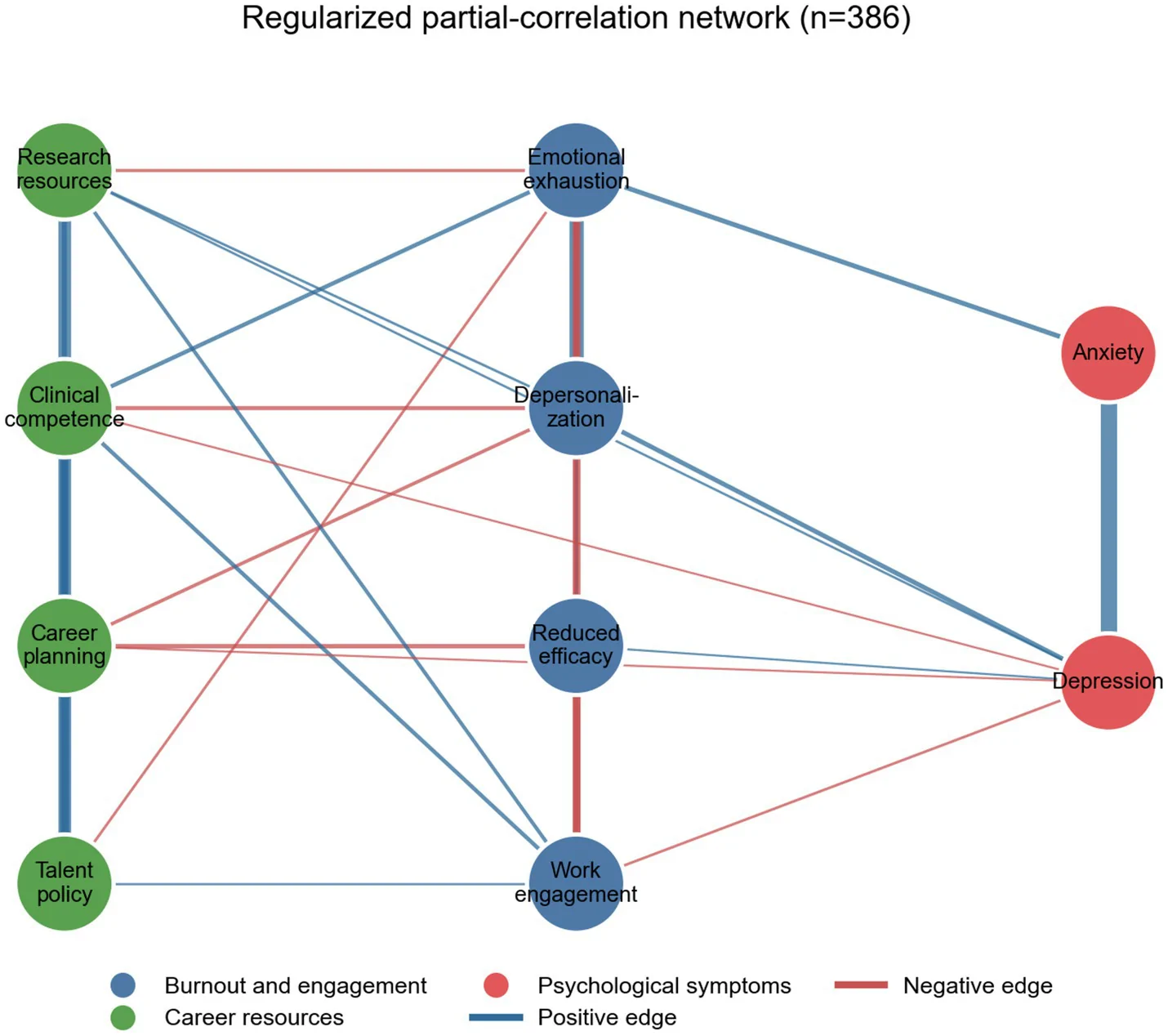
Regularized partial-correlation network. Blue edges are positive and red edges negative; edge width represents absolute regularized partial-correlation magnitude. Node colors identify the prespecified communities. Edges are conditional associations and do not establish causal pathways.

Emotional exhaustion had the highest strength (1.45) and occupied the highest-strength position in 95.90% of nonparametric bootstrap samples. Its pairwise strength difference excluded zero for every node except talent-policy support. The strength correlation-stability coefficient was 0.40, so the ranking was interpreted as moderately stable rather than definitive. In the primary scoring, career planning had the largest-magnitude negative bridge expected influence (−0.28) and occupied the most-negative bridge position in 93.50% of bootstrap samples; its bootstrap interval was −0.40 to −0.13. Bridge expected influence also had a correlation-stability coefficient of 0.40. However, after cp5 was excluded, the most-negative bridge rank shifted from career planning to reduced efficacy. We therefore treated the career-planning bridge result as exploratory and scoring-sensitive (Figure 3 and Supplementary Tables S14, S20). Exploratory permutation tests did not support sex differences in global strength (*p* = 0.778) or maximum edge difference (*p* = 0.599) (Supplementary Table S7 and Supplementary Figure S2).

**Figure 3:**
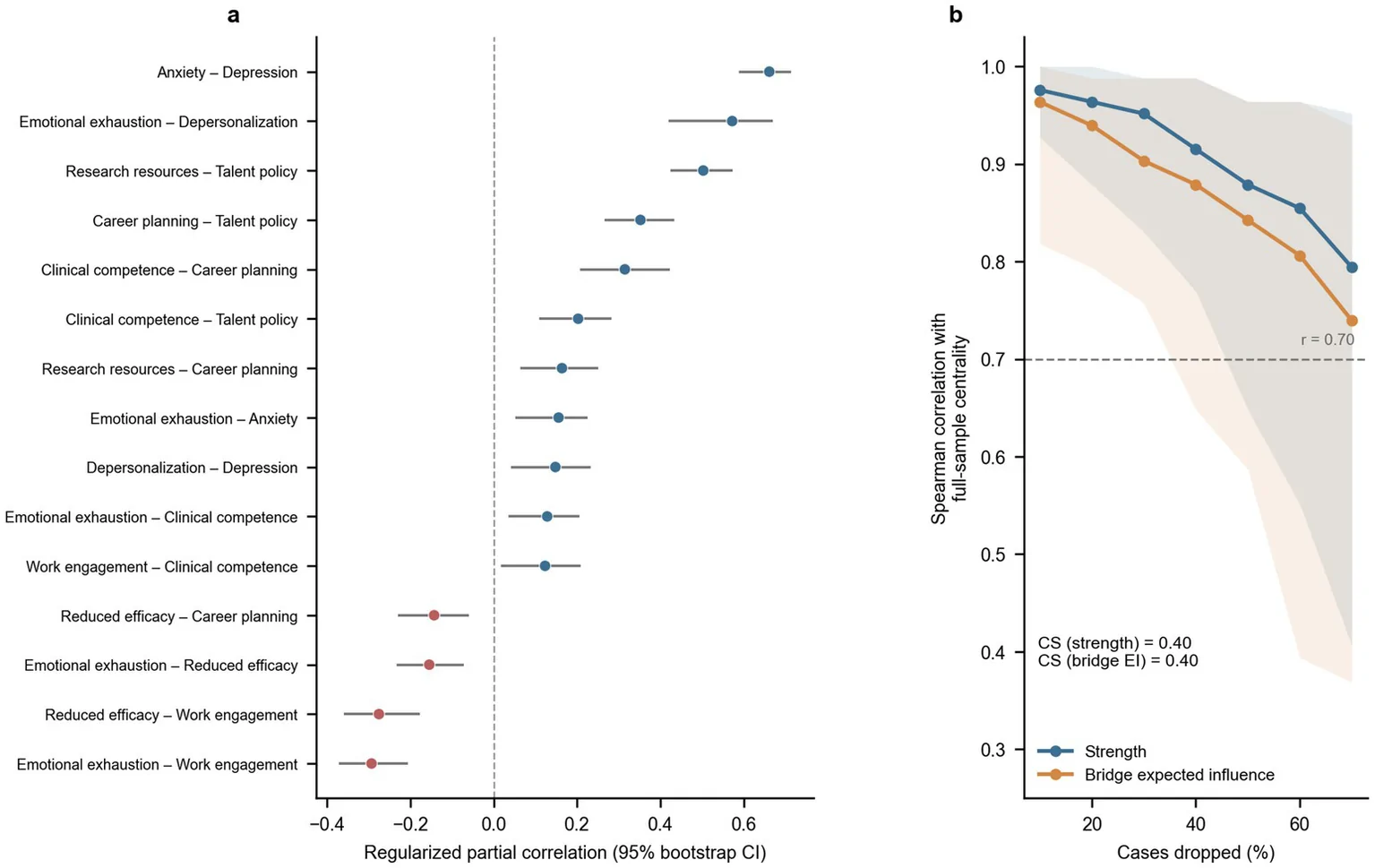
Network accuracy and centrality stability. Panel **(a)** displays the 15 largest estimated edges with 95% confidence intervals from 1,000 non-parametric bootstrap resamples. Panel **(b)** shows case-dropping correlations with full-sample strength and bridge expected influence, based on 200 resamples at each retained fraction. Both correlation-stability coefficients were 0.40. Shaded bands are 95% intervals; *n* = 386.

## Discussion

This study combined LPA and network analysis to examine heterogeneity in burnout and work engagement and their relationships with career resources and psychological symptoms among early-career doctoral healthcare professionals facing concurrent clinical and academic demands. Three profiles were identified: resilient–engaged, moderate, and high-strain. The resilient–engaged profile accounted for only 16.84% of participants, whereas the high-strain profile was the largest (42.75%). The main contribution is not a new diagnostic classification; it is the joint demonstration that occupational strain is heterogeneous, that the profile gradient corresponds to anxiety and depressive symptoms, and that perceived career-development planning is consistently associated with lower-strain membership. This extends early-career workforce research by connecting professional-development resources with multidimensional occupational well-being, operationalized here as burnout, work engagement, and psychological symptoms. The findings are consistent with person-centered studies reporting heterogeneous burnout patterns among hospital physicians and emergency nurses (16).

Career-development planning was the resource most consistently associated with profile membership. From a COR perspective, the observed low-to-high strain gradient is compatible with differing degrees of perceived resource depletion, while clear career guidance may function as a contextual resource that reduces uncertainty about clinical, research, promotion, and mentoring demands (9, 10). Career planning may also help professionals decide where to invest limited time and effort and where additional organizational support is needed. This interpretation is consistent with evidence linking supervision and organizational change to engagement and burnout among physicians (12, 14). However, the study measured neither resource change over time nor a loss spiral. Reverse direction remains plausible: participants with less burnout may evaluate career opportunities more favorably. Longitudinal studies are needed to determine the temporal ordering of career resources, engagement, and strain.

Anxiety and depressive symptoms were more pronounced in the moderate and high-strain profiles, particularly in the high-strain group. This finding is consistent with previous evidence showing close links between burnout, anxiety, and depression among physicians and physicians in training (22). Although the GAD-2 and PHQ-2 are screening rather than diagnostic tools, the concentration of positive screens in the high-strain profile highlights the need for confidential mental-health assessment and voluntary support alongside career-development policies (6).

The network findings indicate that emotional exhaustion was strongly connected to other study variables, consistent with previous healthcare research (33). Its highest-strength position was frequent in bootstrap samples, but its difference from talent-policy support remained uncertain and the centrality CS coefficient was 0.40. Career planning showed the most-negative bridge expected influence in the primary scoring, yet this rank changed after cp5 removal. Bridge expected influence therefore describes a scoring-sensitive pattern of cross-domain conditional connectivity, not a tested mechanism or intervention target (20, 34). By including organizational career resources, the analysis extends earlier networks focused mainly on psychological symptoms or professional efficacy (35). Longitudinal or experimental data are required before concluding that changing any node would alter exhaustion or psychological distress.

No clear sex differences were found in the network structure, but this does not exclude the influence of gender-related factors such as caregiving burden, discrimination, and unequal access to organizational opportunities (36). Future studies should measure these factors directly. For hospital management, the findings support clearer career pathways, protected research time, coordinated clinical–academic supervision, and confidential voluntary mental-health support (37). These measures require prospective evaluation, and profile membership should not be used in employment decisions.

## Limitations

The study has several limitations. First, its cross-sectional, self-report design prevents temporal or causal inference and is vulnerable to common-method variance, recall and social-desirability bias, reverse direction, and residual confounding. Second, purposive recruitment from one tertiary research-oriented hospital, supplemented by referrals within a single Chinese province, did not provide a probability-based sampling frame. The findings may therefore not generalize to doctoral professionals in other regions, hospital types, career stages, or health systems. Third, the locally developed career-resource questionnaire was refined and evaluated in the same sample. Despite good alpha and omega values and favorable ordinal-CFA sensitivity, the maximum-likelihood CFA fit was only acceptable-to-marginal, two retained reverse-worded items had low loadings, and two HTMT values exceeded 0.90. Test–retest reliability, criterion validity, and external cross-validation were not assessed; item-deletion sensitivity reduces but does not remove this measurement limitation. Taken together, these findings indicate that the psychometric properties of the career resource instrument are only marginal, which constitutes a key limitation of this study. Fourth, profile and network results depended on modeling and scoring choices. Information criteria continued to improve as higher-class solutions accumulated boundary variances or very small classes, while strength and bridge stability were moderate and the bridge rank changed after cp5 removal. Finally, GAD-2 and PHQ-2 are brief screening tools and do not establish clinical diagnoses.

## Conclusion

Early-career doctoral healthcare professionals showed a burnout–engagement severity gradient, and the high-strain group reported the greatest anxiety and depressive symptoms. Perceived career-development planning was consistently associated with lower-strain membership, while emotional exhaustion was the most strongly connected node in most bootstrap samples. These cross-sectional findings support further prospective evaluation of clearer career pathways, protected academic time, coordinated mentoring, and confidential mental-health support; they do not establish that these measures will prevent high strain or that any network node is a causal intervention target.

## Data Availability

The datasets presented in this article are not readily available because of privacy and ethical restrictions to protect research participants. Requests to access the datasets should be directed to the corresponding author.
