# Supplementary material for: Burnout and work-engagement profiles, career resources, and psychological distress among early-career doctoral healthcare professionals: a cross-sectional latent profile and network analysis
Source: Front Public Health. 2026 Sep 23;14:1953465. doi: 10.3389/fpubh.2026.1953465 (PMC13642826; doi:10.3389/fpubh.2026.1953465)
Supplement: Supplementary file 1 [file Data_sheet_1.docx]

**Tables and Figures**

**Burnout and work-engagement profiles, career resources, and psychological distress among early-career doctoral healthcare professionals: a cross-sectional latent profile and network analysis**

**Supplementary material: Tables S1–S20 and Figures S1–S2**

Supplementary tables

Supplementary Table S1. Internal consistency.

| **Scale or dimension** | **Items** | **Complete-case N** | **Cronbach alpha** |
| --- | --- | --- | --- |
| Emotional exhaustion | 4 | 386 | 0.94 |
| Depersonalization | 5 | 386 | 0.94 |
| Reduced professional efficacy | 3 | 386 | 0.81 |
| Work engagement | 9 | 386 | 0.93 |
| Job satisfaction | 20 | 386 | 0.97 |
| Research resource support | 5 | 386 | 0.87 |
| Clinical competence enhancement | 4 | 380 | 0.88 |
| Career development planning | 5 | 386 | 0.83 |
| Talent policy support | 5 | 386 | 0.86 |
| GAD-2 | 2 | 386 | 0.88 |
| PHQ-2 | 2 | 386 | 0.85 |

Supplementary Table S2. Confirmatory factor-analysis fit.

| **Model** | **N** | **Chi-square** | **df** | **Chi-square/df** | **CFI** | **TLI** | **RMSEA** |
| --- | --- | --- | --- | --- | --- | --- | --- |
| Occupational burnout (three-factor) | 386 | 180.03 | 51 | 3.53 | 0.97 | 0.96 | 0.08 |
| Career development resources (four-factor) | 380 | 571.64 | 146 | 3.92 | 0.92 | 0.91 | 0.09 |

**Note.** Career-resource CFA used complete cases. Fit estimates describe internal structure in this sample and do not constitute external validation.

Supplementary Table S3. Standardized factor loadings.

| **Scale** | **Item code** | **Factor** | **Standardized loading** | **P value** |
| --- | --- | --- | --- | --- |
| Occupational burnout (three-factor) | ee1 | EE | 0.92 | — |
| Occupational burnout (three-factor) | ee2 | EE | 0.90 | <0.001 |
| Occupational burnout (three-factor) | ee3 | EE | 0.90 | <0.001 |
| Occupational burnout (three-factor) | ee4 | EE | 0.84 | <0.001 |
| Occupational burnout (three-factor) | dp1 | DP | 0.91 | — |
| Occupational burnout (three-factor) | dp2 | DP | 0.94 | <0.001 |
| Occupational burnout (three-factor) | dp3 | DP | 0.90 | <0.001 |
| Occupational burnout (three-factor) | dp4 | DP | 0.79 | <0.001 |
| Occupational burnout (three-factor) | dp5 | DP | 0.86 | <0.001 |
| Occupational burnout (three-factor) | rpe1 | RPE | 0.72 | — |
| Occupational burnout (three-factor) | rpe2 | RPE | 0.77 | <0.001 |
| Occupational burnout (three-factor) | rpe3 | RPE | 0.81 | <0.001 |
| Career development resources (four-factor) | rs1 | RS | 0.81 | — |
| Career development resources (four-factor) | rs2 | RS | 0.87 | <0.001 |
| Career development resources (four-factor) | rs3 | RS | 0.88 | <0.001 |
| Career development resources (four-factor) | rs4 | RS | 0.90 | <0.001 |
| Career development resources (four-factor) | rs5 | RS | 0.38 | <0.001 |
| Career development resources (four-factor) | cc1 | CC | 0.89 | — |
| Career development resources (four-factor) | cc2 | CC | 0.88 | <0.001 |
| Career development resources (four-factor) | cc3 | CC | 0.70 | <0.001 |
| Career development resources (four-factor) | cc4 | CC | 0.77 | <0.001 |
| Career development resources (four-factor) | cp1 | CP | 0.85 | — |
| Career development resources (four-factor) | cp2 | CP | 0.80 | <0.001 |
| Career development resources (four-factor) | cp3 | CP | 0.83 | <0.001 |
| Career development resources (four-factor) | cp4 | CP | 0.85 | <0.001 |
| Career development resources (four-factor) | cp5 | CP | 0.35 | <0.001 |
| Career development resources (four-factor) | tp1 | TP | 0.79 | — |
| Career development resources (four-factor) | tp2 | TP | 0.89 | <0.001 |
| Career development resources (four-factor) | tp3 | TP | 0.83 | <0.001 |
| Career development resources (four-factor) | tp4 | TP | 0.84 | <0.001 |
| Career development resources (four-factor) | tp5 | TP | 0.44 | <0.001 |

**Note.** Table S3 reports the final four-factor CFA after the clinical-pressure item had been excluded during questionnaire refinement; that item was not included in the final domain score or CFA. Within the retained item set, the reverse-worded research-resource item rs5 and career-planning item cp5 had standardized loadings of 0.38 and 0.35, respectively. They were retained because item-retention decisions considered prespecified content coverage in addition to loading magnitude; these item-level results and the locally developed domain scores should therefore be interpreted cautiously and externally validated. Item wording for the adapted burnout measure is not reproduced because of licensing considerations.

Supplementary Table S4. Node centrality.

| **Node** | **Community** | **Strength** | **Expected influence** | **Bridge expected influence** |
| --- | --- | --- | --- | --- |
| Emotional exhaustion | Burnout and engagement | 1.45 | 0.26 | 0.14 |
| Depersonalization | Burnout and engagement | 1.17 | 0.50 | -0.02 |
| Reduced efficacy | Burnout and engagement | 0.69 | -0.50 | -0.13 |
| Work engagement | Burnout and engagement | 1.03 | -0.45 | 0.23 |
| Research resources | Career resources | 0.96 | 0.79 | 0.13 |
| Clinical competence | Career resources | 0.95 | 0.59 | 0.08 |
| Career planning | Career resources | 1.15 | 0.56 | -0.28 |
| Talent policy | Career resources | 1.17 | 1.02 | -0.03 |
| Anxiety | Psychological symptoms | 0.84 | 0.79 | 0.13 |
| Depression | Psychological symptoms | 1.03 | 0.75 | 0.09 |

Supplementary Table S5. Regularized network edges and bootstrap intervals.

| **Node 1** | **Node 2** | **Weight** | **Bootstrap mean** | **95% CI low** | **95% CI high** | **CI excludes zero** |
| --- | --- | --- | --- | --- | --- | --- |
| Emotional exhaustion | Depersonalization | 0.57 | 0.56 | 0.42 | 0.67 | Yes |
| Emotional exhaustion | Reduced efficacy | -0.15 | -0.16 | -0.23 | -0.07 | Yes |
| Emotional exhaustion | Work engagement | -0.29 | -0.29 | -0.37 | -0.21 | Yes |
| Emotional exhaustion | Research resources | -0.08 | -0.09 | -0.18 | 0.00 | No |
| Emotional exhaustion | Clinical competence | 0.13 | 0.13 | 0.04 | 0.21 | Yes |
| Emotional exhaustion | Career planning | 0.00 | -0.01 | -0.09 | 0.04 | No |
| Emotional exhaustion | Talent policy | -0.07 | -0.05 | -0.13 | 0.00 | No |
| Emotional exhaustion | Anxiety | 0.16 | 0.14 | 0.05 | 0.23 | Yes |
| Emotional exhaustion | Depression | 0.00 | 0.02 | -0.05 | 0.10 | No |
| Depersonalization | Reduced efficacy | 0.06 | 0.07 | 0.00 | 0.15 | No |
| Depersonalization | Work engagement | -0.11 | -0.12 | -0.26 | 0.00 | Yes |
| Depersonalization | Research resources | 0.06 | 0.05 | 0.00 | 0.13 | No |
| Depersonalization | Clinical competence | -0.12 | -0.13 | -0.22 | -0.04 | Yes |
| Depersonalization | Career planning | -0.10 | -0.09 | -0.16 | 0.00 | Yes |
| Depersonalization | Talent policy | 0.00 | -0.01 | -0.07 | 0.03 | No |
| Depersonalization | Anxiety | 0.00 | 0.02 | -0.04 | 0.11 | No |
| Depersonalization | Depression | 0.15 | 0.14 | 0.04 | 0.23 | Yes |
| Reduced efficacy | Work engagement | -0.28 | -0.27 | -0.36 | -0.18 | Yes |
| Reduced efficacy | Research resources | 0.00 | -0.01 | -0.08 | 0.05 | No |
| Reduced efficacy | Clinical competence | -0.02 | -0.03 | -0.11 | 0.03 | No |
| Reduced efficacy | Career planning | -0.14 | -0.14 | -0.23 | -0.06 | Yes |
| Reduced efficacy | Talent policy | 0.00 | 0.01 | -0.05 | 0.10 | No |
| Reduced efficacy | Anxiety | 0.00 | 0.00 | -0.04 | 0.04 | No |
| Reduced efficacy | Depression | 0.03 | 0.04 | 0.00 | 0.10 | No |
| Work engagement | Research resources | 0.11 | 0.10 | 0.00 | 0.20 | No |
| Work engagement | Clinical competence | 0.12 | 0.11 | 0.02 | 0.21 | Yes |
| Work engagement | Career planning | 0.02 | 0.03 | -0.01 | 0.10 | No |
| Work engagement | Talent policy | 0.04 | 0.05 | 0.00 | 0.14 | No |
| Work engagement | Anxiety | 0.00 | -0.01 | -0.08 | 0.06 | No |
| Work engagement | Depression | -0.06 | -0.05 | -0.14 | 0.00 | No |
| Research resources | Clinical competence | 0.00 | 0.01 | -0.05 | 0.08 | No |
| Research resources | Career planning | 0.16 | 0.16 | 0.06 | 0.25 | Yes |
| Research resources | Talent policy | 0.50 | 0.50 | 0.42 | 0.57 | Yes |
| Research resources | Anxiety | 0.00 | 0.01 | -0.04 | 0.06 | No |
| Research resources | Depression | 0.05 | 0.05 | 0.00 | 0.13 | No |
| Clinical competence | Career planning | 0.31 | 0.31 | 0.21 | 0.42 | Yes |
| Clinical competence | Talent policy | 0.20 | 0.20 | 0.11 | 0.28 | Yes |
| Clinical competence | Anxiety | 0.00 | 0.01 | -0.03 | 0.07 | No |
| Clinical competence | Depression | -0.04 | -0.04 | -0.12 | 0.00 | No |
| Career planning | Talent policy | 0.35 | 0.35 | 0.27 | 0.43 | Yes |
| Career planning | Anxiety | -0.02 | -0.02 | -0.08 | 0.01 | No |
| Career planning | Depression | -0.03 | -0.03 | -0.10 | 0.00 | No |
| Talent policy | Anxiety | 0.00 | -0.01 | -0.06 | 0.03 | No |
| Talent policy | Depression | -0.01 | -0.01 | -0.07 | 0.02 | No |
| Anxiety | Depression | 0.66 | 0.66 | 0.59 | 0.71 | Yes |

Supplementary Table S6. Case-dropping stability of node strength.

| **Retained** | **Dropped** | **Median correlation** | **95% CI low** | **95% CI high** | **Pr (r≥0.70)** |
| --- | --- | --- | --- | --- | --- |
| 90% | 10% | 0.98 | 0.93 | 1.00 | 1.00 |
| 80% | 20% | 0.96 | 0.88 | 1.00 | 1.00 |
| 70% | 30% | 0.95 | 0.79 | 0.99 | 1.00 |
| 60% | 40% | 0.93 | 0.76 | 0.99 | 0.99 |
| 50% | 50% | 0.89 | 0.69 | 0.98 | 0.94 |
| 40% | 60% | 0.84 | 0.56 | 0.96 | 0.87 |
| 30% | 70% | 0.76 | 0.39 | 0.96 | 0.66 |

Supplementary Table S7. Exploratory sex-stratified network permutation tests.

| **Test** | **Observed difference** | **P value** | **Permutations** |
| --- | --- | --- | --- |
| Global strength difference | 0.10 | 0.778 | 1000 |
| Maximum edge difference | 0.19 | 0.599 | 1000 |

**Note.** Neither test supported a sex difference in network structure.

Supplementary Table S8. LPA fit and boundary-variance diagnostics.

| **Classes** | **AIC** | **BIC** | **SABIC** | **Entropy** | **Mean max posterior** | **Smallest class** | **Boundary variances** |
| --- | --- | --- | --- | --- | --- | --- | --- |
| 1 | 4397.70 | 4429.30 | 4403.90 | - | 1.00 | 386 (100.00%) | 0 |
| 2 | 3832.80 | 3900.00 | 3846.10 | 0.82 | 0.95 | 184 (47.67%) | 0 |
| 3 | 2781.10 | 2884.00 | 2801.50 | 0.92 | 0.97 | 65 (16.84%) | 1 |
| 4 | 2072.40 | 2210.80 | 2099.80 | 0.96 | 0.98 | 65 (16.84%) | 2 |
| 5 | 1769.50 | 1943.50 | 1803.90 | 0.96 | 0.97 | 21 (5.44%) | 3 |
| 6 | 879.50 | 1089.20 | 921.00 | 0.97 | 0.98 | 5 (1.30%) | 7 |

**Note.** Primary class-specific diagonal-covariance models were refitted with 500 random starts and reg_covar=1e-6. Boundary variances are class-specific standardized variance estimates at the numerical lower bound. The accumulation of boundary estimates indicates that the large information-criterion improvements in higher-class solutions partly reflected floor-level or near-degenerate components. Entropy is not applicable to a one-class model.

Supplementary Table S9. Three- and four-class profile means.

| **Classes** | **Ordered profile** | **n** | **EE mean (SD)** | **DP mean (SD)** | **RPE mean (SD)** | **WE mean (SD)** |
| --- | --- | --- | --- | --- | --- | --- |
| 3 | Class 1 | 65 | 1.22 (0.34) | 1.00 (0.00) | 1.85 (1.29) | 4.73 (0.31) |
| 3 | Class 2 | 156 | 2.41 (0.50) | 1.84 (0.33) | 2.17 (0.67) | 4.02 (0.41) |
| 3 | Class 3 | 165 | 3.50 (0.63) | 3.00 (0.62) | 2.67 (0.54) | 3.20 (0.55) |
| 4 | Class 1 | 65 | 1.22 (0.34) | 1.00 (0.00) | 1.85 (1.29) | 4.73 (0.31) |
| 4 | Class 2 | 78 | 2.45 (0.54) | 1.67 (0.38) | 1.90 (0.40) | 4.12 (0.41) |
| 4 | Class 3 | 74 | 2.40 (0.50) | 2.00 (0.00) | 2.41 (0.76) | 3.91 (0.42) |
| 4 | Class 4 | 169 | 3.46 (0.65) | 2.98 (0.63) | 2.68 (0.55) | 3.22 (0.56) |

**Note.** Profiles are ordered from lower to higher strain. All scores are item means on a 1-5 scale; higher RPE indicates lower perceived efficacy. The four-class model retained the low- and high-strain states and divided the intermediate band into two groups with overlapping EE and WE means.

Supplementary Table S10. LPA random-start and variance-specification sensitivity.

| **Variance specification** | **Classes** | **BIC** | **Entropy** | **Smallest class** | **Median ARI** | **Starts at best LL** | **ARI vs primary 3-class** |
| --- | --- | --- | --- | --- | --- | --- | --- |
| Primary diagonal | 3 | 2884.00 | 0.92 | 65 (16.84%) | 1.00 | 74.00% | 1.00 |
| Primary diagonal | 4 | 2210.80 | 0.96 | 65 (16.84%) | 1.00 | 68.00% | - |
| Equal diagonal | 3 | 3798.60 | 0.86 | 76 (19.69%) | - | - | 0.75 |
| Equal diagonal | 4 | 3633.00 | 0.94 | 14 (3.63%) | - | - | - |

**Note.** Random-start diagnostics used 100 additional single-start fits for each primary model. ‘Starts at best LL’ is the percentage within 0.01 log-likelihood units of the 100-start solution. Equal-diagonal models constrained each indicator variance to be equal across classes. ARI, adjusted Rand index; LL, log likelihood.

Supplementary Table S11. Four-class profile-regression sensitivity.

| **Outcome or predictor** | **Metric** | **Class 2 vs 1** | **Class 3 vs 1** | **Class 4 vs 1** |
| --- | --- | --- | --- | --- |
| Research resources | OR | 0.95 (0.47-1.94); P=0.896 | 0.90 (0.43-1.91); P=0.794 | 0.87 (0.39-1.92); P=0.721 |
| Career planning | OR | 0.19 (0.08-0.44); P<0.001 | 0.38 (0.17-0.83); P=0.015 | 0.14 (0.06-0.32); P<0.001 |
| GAD-2 | Beta | 0.82 (0.40-1.24); P<0.001 | 0.78 (0.35-1.20); P<0.001 | 1.62 (1.14-2.10); P<0.001 |
| PHQ-2 | Beta | 0.49 (0.15-0.83); P=0.005 | 0.53 (0.16-0.89); P=0.005 | 1.32 (0.88-1.76); P<0.001 |

**Note.** Resource rows are from the fully adjusted multinomial model and report ORs per SD increase. GAD-2 and PHQ-2 rows report fully adjusted mean differences from robust linear models. Values are estimate (95% CI). Class 1 was the resilient-engaged reference; classes 2 and 3 were adjacent intermediate groups; class 4 was high-strain.

Supplementary Table S12. Low-loading-item deletion: scale properties and CFA fit.

| **Scoring scenario** | **RS alpha** | **CP alpha** | **RS score r** | **CP score r** | **CFI** | **TLI** | **RMSEA** |
| --- | --- | --- | --- | --- | --- | --- | --- |
| Primary 19-item scoring | 0.87 | 0.83 | 1.00 | 1.00 | 0.92 | 0.91 | 0.09 |
| Exclude rs5 | 0.92 | 0.83 | 0.97 | 1.00 | 0.94 | 0.92 | 0.09 |
| Exclude cp5 | 0.87 | 0.90 | 1.00 | 0.96 | 0.93 | 0.92 | 0.09 |
| Exclude rs5 and cp5 | 0.92 | 0.90 | 0.97 | 0.96 | 0.94 | 0.93 | 0.08 |

**Note.** The primary questionnaire retained rs5 and cp5 for prespecified content coverage. Sensitivity scenarios excluded the retained low-loading items separately or jointly. Score r is the Pearson correlation with the primary domain score. RS, research resources; CP, career planning.

Supplementary Table S13. Low-loading-item deletion: adjusted profile regression.

| **Scoring scenario** | **Comparison** | **Research resources**  **OR (95% CI)** | **Career planning**  **OR (95% CI)** |
| --- | --- | --- | --- |
| Primary 19-item scoring | Moderate vs resilient-engaged | 0.97 (0.49-1.92); P=0.930 | 0.26 (0.13-0.52); P<0.001 |
| Primary 19-item scoring | High-strain vs resilient-engaged | 0.75 (0.35-1.64); P=0.478 | 0.16 (0.07-0.36); P<0.001 |
| Exclude rs5 | Moderate vs resilient-engaged | 1.27 (0.66-2.44); P=0.467 | 0.24 (0.12-0.49); P<0.001 |
| Exclude rs5 | High-strain vs resilient-engaged | 1.18 (0.54-2.56); P=0.680 | 0.15 (0.07-0.33); P<0.001 |
| Exclude cp5 | Moderate vs resilient-engaged | 0.90 (0.45-1.80); P=0.766 | 0.38 (0.16-0.88); P=0.024 |
| Exclude cp5 | High-strain vs resilient-engaged | 0.66 (0.31-1.44); P=0.301 | 0.33 (0.14-0.82); P=0.017 |
| Exclude rs5 and cp5 | Moderate vs resilient-engaged | 1.31 (0.68-2.52); P=0.419 | 0.32 (0.14-0.75); P=0.009 |
| Exclude rs5 and cp5 | High-strain vs resilient-engaged | 1.15 (0.53-2.47); P=0.726 | 0.28 (0.11-0.68); P=0.005 |

**Note.** ORs are per SD increase from the same fully adjusted multinomial model used in the primary analysis. Career-planning associations remained statistically supported in every scoring scenario, although effect sizes were attenuated after cp5 exclusion.

Supplementary Table S14. Low-loading-item deletion: network sensitivity.

| **Scoring scenario** | **Edge-weight r** | **Maximum edge difference** | **CP bridge EI** | **EE strength** | **Highest-strength node** | **Most-negative bridge node** |
| --- | --- | --- | --- | --- | --- | --- |
| Primary 19-item scoring | 1.00 | 0.00 | -0.28 | 1.45 | Emotional exhaustion | Career planning |
| Exclude rs5 | 1.00 | 0.07 | -0.28 | 1.43 | Emotional exhaustion | Career planning |
| Exclude cp5 | 0.99 | 0.10 | -0.09 | 1.44 | Emotional exhaustion | Reduced efficacy |
| Exclude rs5 and cp5 | 0.99 | 0.09 | -0.10 | 1.40 | Emotional exhaustion | Reduced efficacy |

**Note.** Edge-weight r compares all 45 regularized partial-correlation weights with the primary network. The broader edge pattern and the highest-strength node were stable, but the exact most-negative bridge rank changed after cp5 exclusion. EI, expected influence; EE, emotional exhaustion; CP, career planning.

Supplementary Table S15. Career-development resource questionnaire items and scoring key.

| **Item** | **Domain** | **English item wording** | **Reverse-scored** |
| --- | --- | --- | --- |
| rs1 | Research resources | The hospital provides sufficient start-up funding for my research. | No |
| rs2 | Research resources | The hospital's equipment and platforms meet my research needs. | No |
| rs3 | Research resources | I can readily obtain the clinical samples or data needed for my research. | No |
| rs4 | Research resources | A mature research team in my department provides support. | No |
| rs5 | Research resources | I lack sufficient time to devote to research work. | Yes |
| cc1 | Clinical competence | The hospital provides systematic clinical supervision and training. | No |
| cc2 | Clinical competence | My clinical procedural skills have improved rapidly. | No |
| cc3 | Clinical competence | I can communicate effectively with patients and their family members. | No |
| cc4 | Clinical competence | Multidisciplinary collaboration mechanisms support my clinical work. | No |
| cp1 | Career planning | The hospital provides clear career-development guidance. | No |
| cp2 | Career planning | I am clear about my professional positioning (clinical, research, or combined). | No |
| cp3 | Career planning | Promotion policies are fair and reasonable for doctoral staff. | No |
| cp4 | Career planning | I have a clear career-development plan for the next 3-5 years. | No |
| cp5 | Career planning | I feel that my opportunities for career development are limited. | Yes |
| tp1 | Talent policy | The hospital's recruitment package (e.g., housing or settlement support) is attractive. | No |
| tp2 | Talent policy | The hospital provides sufficient opportunities for academic exchange. | No |
| tp3 | Talent policy | My compensation is commensurate with my work contribution. | No |
| tp4 | Talent policy | The hospital values the training and development of early-career doctoral staff. | No |
| tp5 | Talent policy | Overall, I am dissatisfied with the hospital's talent-support policies. | Yes |

**Note.** Items were rated from 1 to 5; reverse-worded items were recoded so that higher domain scores consistently indicated greater resources. The original clinical-pressure item was excluded during questionnaire refinement and is not listed. English wording is a study translation of the Chinese questionnaire.

Supplementary Table S16. LPA parameter specification and model-selection decision matrix.

| **Variance specification** | **k** | **Free parameters** | **BIC** | **Entropy** | **Mean max posterior** | **Smallest class** | **Boundary variances** | **Decision** |
| --- | --- | --- | --- | --- | --- | --- | --- | --- |
| Class-specific | 1 | 8 | 4429.30 | - | 1.00 | 386 (100.00%) | 0 | Under-extracted |
| Class-specific | 2 | 17 | 3900.00 | 0.82 | 0.95 | 184 (47.67%) | 0 | Under-extracted |
| Class-specific | 3 | 26 | 2884.00 | 0.92 | 0.97 | 65 (16.84%) | 1 | Retained: first adequate low/moderate/high solution |
| Class-specific | 4 | 35 | 2210.80 | 0.96 | 0.98 | 65 (16.84%) | 2 | Boundary growth; redundant intermediate split |
| Class-specific | 5 | 44 | 1943.50 | 0.96 | 0.97 | 21 (5.44%) | 3 | Boundary growth; adjacent/floor split |
| Class-specific | 6 | 53 | 1089.20 | 0.97 | 0.98 | 5 (1.30%) | 7 | Fails minimum class size |
| Equal diagonal | 2 | 13 | 3960.30 | 0.83 | 0.95 | 180 (46.63%) | 0 | Under-extracted |
| Equal diagonal | 3 | 18 | 3798.60 | 0.86 | 0.94 | 76 (19.69%) | 0 | Sensitivity: preserves three-level structure |
| Equal diagonal | 4 | 23 | 3633.00 | 0.94 | 0.97 | 14 (3.63%) | 0 | Fails minimum class size |
| Equal diagonal | 5 | 28 | 3471.93 | 0.93 | 0.96 | 10 (2.59%) | 0 | Fails minimum class size |
| Equal diagonal | 6 | 33 | 3424.06 | 0.95 | 0.96 | 1 (0.26%) | 0 | Fails minimum class size |

**Note.** The primary class-specific diagonal models used 500 random starts; equal-diagonal sensitivity models used 200. All models converged. The variance lower bound was 1e-6. Decision aids were entropy and mean maximum posterior probability ≥0.80 and a smallest class ≥5% and n≥20; these were considered with the fit trajectory, boundary estimates, stability, parsimony, and interpretability and were not formal hypothesis tests. The three-class primary solution was retained because it was the first interpretable low/moderate/high-strain solution meeting the classification and size aids. The equal-diagonal three-class solution agreed substantially with it (adjusted Rand index=0.75), whereas the four-, five-, and six-class constrained solutions contained only 14, 10, and 1 participant in their smallest classes. BIC was not used in isolation because successive reductions accompanied boundary estimates, redundant adjacent-profile splits, or inadmissibly small classes.

**Supplementary Table S17. Classification-quality diagnostics for the retained three-class model.**

| **Assigned profile** | **n (%)** | **Average posterior probability** | **Median posterior probability** | **Posterior probability ≥0.80** | **Odds of correct classification** |
| --- | --- | --- | --- | --- | --- |
| Resilient-engaged | 65 (16.84%) | 1.00 | 1.00 | 100.00% | 11662.40 |
| Moderate | 156 (40.41%) | 0.95 | 0.99 | 92.95% | 30.60 |
| High-strain | 165 (42.75%) | 0.96 | 1.00 | 91.52% | 35.40 |

**Note.** Posterior summaries are calculated within the modal assigned class. The sample-average maximum posterior probability was 0.97, and the estimated modal-classification error was 3.40%. Together, these values indicate good overall separation. Table S18 examines whether the remaining assignment uncertainty changes the regression estimates.

**Supplementary Table S18. Regression sensitivity to latent-profile classification uncertainty.**

| **Model target** | **Comparison** | **Predictor or contrast** | **Modal assignment estimate**  **(95% CI)** | **Posterior-probability estimate**  **(95% CI)** |
| --- | --- | --- | --- | --- |
| Profile membership | Moderate vs resilient-engaged | Research resources (per SD) | OR 0.97 (0.49-1.92); P=0.930 | OR 0.99 (0.50-1.96); P=0.983 |
| Profile membership | High-strain vs resilient-engaged | Research resources (per SD) | OR 0.75 (0.35-1.64); P=0.478 | OR 0.75 (0.34-1.66); P=0.483 |
| Profile membership | Moderate vs resilient-engaged | Career planning (per SD) | OR 0.26 (0.13-0.52); P<0.001 | OR 0.26 (0.13-0.53); P<0.001 |
| Profile membership | High-strain vs resilient-engaged | Career planning (per SD) | OR 0.16 (0.07-0.36); P<0.001 | OR 0.16 (0.07-0.37); P<0.001 |
| GAD-2 | Moderate vs resilient-engaged | Adjusted profile difference | Beta 0.81 (0.43-1.18); P<0.001 | Beta 0.79 (0.42-1.16); P<0.001 |
| GAD-2 | High-strain vs resilient-engaged | Adjusted profile difference | Beta 1.65 (1.16-2.14); P<0.001 | Beta 1.66 (1.17-2.15); P<0.001 |
| PHQ-2 | Moderate vs resilient-engaged | Adjusted profile difference | Beta 0.52 (0.20-0.83); P=0.001 | Beta 0.50 (0.18-0.82); P=0.002 |
| PHQ-2 | High-strain vs resilient-engaged | Adjusted profile difference | Beta 1.35 (0.90-1.80); P<0.001 | Beta 1.32 (0.87-1.78); P<0.001 |

**Note.** Posterior-probability estimates combine 500 pseudo-class datasets using Rubin's rules. Each participant's class was drawn from the fitted three-class posterior-probability vector in every dataset. Multinomial models used the resilient-engaged profile as reference and the same fully adjusted covariates as the primary analysis; distress models used the same robust adjusted specifications. Estimates from the two approaches were similar, indicating that residual classification uncertainty did not materially change the conclusions.

Supplementary Table S19A. Career-resource reliability and factor-loading sensitivity.

| **Domain** | **Items** | **Cronbach α** | **McDonald ω** | **ML loading range** | **Ordinal DWLS loading range** |
| --- | --- | --- | --- | --- | --- |
| Research resources | 5 | 0.87 | 0.91 | 0.38–0.90 | 0.38–0.93 |
| Clinical competence | 4 | 0.88 | 0.91 | 0.70–0.89 | 0.74–0.92 |
| Career planning | 5 | 0.83 | 0.90 | 0.35–0.85 | 0.41–0.92 |
| Talent policy | 5 | 0.86 | 0.90 | 0.44–0.89 | 0.52–0.92 |

Note. The ordinal polychoric DWLS model used 380 complete cases and yielded χ²=104.43, df=146, CFI=1.00, TLI=1.00, and RMSEA=0.00. Improved global fit did not remove the low-loading reverse-worded items. ML, maximum likelihood; DWLS, diagonally weighted least squares.

Supplementary Table S19B. Heterotrait–monotrait ratios for career-resource domains.

| **Domain 1** | **Domain 2** | **HTMT** |
| --- | --- | --- |
| Research resources | Clinical competence | 0.66 |
| Research resources | Career planning | 0.83 |
| Research resources | Talent policy | 0.93 |
| Clinical competence | Career planning | 0.84 |
| Clinical competence | Talent policy | 0.79 |
| Career planning | Talent policy | 0.93 |

Note. HTMT values above 0.90 for research resources versus talent policy (0.93) and career planning versus talent policy (0.93) indicate limited discriminant validity for these locally developed domains. HTMT, heterotrait–monotrait ratio.

Supplementary Table S20. Bootstrap rank uncertainty and bridge stability.

| **Node** | **Strength** | **P(highest strength)** | **EE-minus-node strength 95% CI** | **Bridge EI** | **P(most-negative bridge)** | **Bridge EI 95% CI** |
| --- | --- | --- | --- | --- | --- | --- |
| Emotional exhaustion | 1.45 | 95.90% | Reference | 0.14 | 0.00% | 0.01 to 0.24 |
| Depersonalization | 1.17 | 0.70% | 0.02 to 0.50 | -0.02 | 0.40% | -0.16 to 0.14 |
| Reduced efficacy | 0.69 | 0.00% | 0.46 to 0.92 | -0.13 | 4.20% | -0.27 to -0.01 |
| Work engagement | 1.03 | 0.00% | 0.18 to 0.66 | 0.23 | 0.00% | 0.08 to 0.36 |
| Research resources | 0.96 | 0.00% | 0.20 to 0.67 | 0.13 | 0.00% | -0.06 to 0.29 |
| Clinical competence | 0.94 | 0.00% | 0.26 to 0.65 | 0.08 | 0.60% | -0.15 to 0.22 |
| Career planning | 1.15 | 0.70% | 0.07 to 0.54 | -0.27 | 93.50% | -0.40 to -0.13 |
| Talent policy | 1.17 | 2.70% | -0.01 to 0.47 | -0.03 | 1.30% | -0.15 to 0.14 |
| Anxiety | 0.84 | 0.00% | 0.29 to 0.76 | 0.13 | 0.00% | -0.03 to 0.29 |
| Depression | 1.03 | 0.00% | 0.16 to 0.64 | 0.09 | 0.00% | -0.09 to 0.29 |

Note. Probabilities and intervals are based on 1,000 nonparametric bootstrap samples. The strength and bridge-EI case-dropping correlation-stability coefficients were both 0.40 (200 resamples per retained fraction). Emotional exhaustion had higher strength than all nodes except talent-policy support at the 95% bootstrap-difference level. Career planning was usually the most-negative bridge, but this rank changed after cp5 deletion (Table S14); its exact rank remains exploratory. EE, emotional exhaustion; EI, expected influence.

**Supplementary figures**

**Supplementary Figure S1.** Pearson correlation matrix of the ten core variables.

**Supplementary Figure S2.** Exploratory sex-stratified regularized networks. Formal permutation tests did not support differences in global strength or maximum edge weight.
